# Increased Production of B-Cell Activating Cytokines and Altered Peripheral B-Cell Subset Distribution during HIV-Related Classical Hodgkin Lymphoma

**DOI:** 10.3390/cancers14010128

**Published:** 2021-12-28

**Authors:** Raphael Lievin, Houria Hendel-Chavez, Aliou Baldé, Rémi Lancar, Michèle Algarte-Génin, Roman Krzysiek, Dominique Costagliola, Lambert Assoumou, Yassine Taoufik, Caroline Besson

**Affiliations:** 1 Department of Hematology and Oncology, Hospital of Versailles, 78150 Le Chesnay, France; raphael.lievin@aphp.fr; 2 Service d’Hématologie et Immunologie Biologique, Hôpital Bicêtre, Assistance Publique-Hôpitaux de Paris, 94270 Le Kremlin-Bicêtre, France; houria.chavez@aphp.fr (H.H.-C.); roman.krzysiek@aphp.fr (R.K.); yassine.taoufik@aphp.fr (Y.T.); 3 INSERM 1186, Institut Gustave Roussy, 94805 Villejuif, France; 4 Institut Pierre Louis d’Epidémiologie et de Santé Publique, IPLESP, Sorbonne Université, INSERM UMR-S 1136, 75646 Paris, France; aliou.balde@iplesp.upmc.fr (A.B.); rlancar@yahoo.se (R.L.); michele.genin@iplesp.upmc.fr (M.A.-G.); dominique.costagliola@iplesp.upmc.fr (D.C.); lambert.assoumou@iplesp.upmc.fr (L.A.); 5 Université Versailles Saint Quentin en Yvelines, Université Paris-Saclay, 78180 Montigny le Bretonneux, France; 6 Centre for Research in Epidemiology and Population Health, INSERM Unit 1018, 94800 Villejuif, France

**Keywords:** Hodgkin lymphoma, HIV, immunology, cytokines

## Abstract

**Simple Summary:**

Patients with HIV are at high risk of developing Hodgkin’s lymphoma. This is potentially due to alterations in blood circulating B-lymphocytes and their activating cytokines. We analyzed the distribution of circulating B-lymphocytes and the level of the activating cytokines IL6, IL10 and BAFF in 38 patients with HIV-related Hodgkin’s lymphoma during a 2-year follow-up. We also compared their characteristics at diagnosis with (1) pre-diagnosis serum samples and (2) samples from control HIV-infected subjects without lymphoma. We found an increase in activating cytokines in cases compared to controls. The level of activating cytokines increased in advanced lymphoma. It decreased over time during follow-up. B-lymphocytic count was similar between patients and controls, but their subset distribution differed. There was an overrepresentation of naive B-lymphocytes over memory B-lymphocytes in HIV-associated Hodgkin lymphoma patients, more pronounced in those with advanced lymphoma. Follow-up showed an increase in B-lymphocytic count with an even greater proportion of naive B-cells. Together this suggests that in HIV-infected patients, Hodgkin lymphoma is associated with an altered blood distribution of B-lymphocytic subsets and an increased production of activating cytokines. This environment may contribute to the process of tumorigenesis.

**Abstract:**

Classical Hodgkin Lymphoma incidence increases in HIV-1-infected patients (HIV-cHL). HIV infection is associated with higher B-cell activation. Here, in 38 HIV-cHL patients from the French cohort ANRS-CO16 Lymphovir, we examined longitudinally over 24 months the serum levels of the B-cell activating cytokines IL10, IL6, and BAFF, and blood distribution of B-cell subsets. Fourteen HIV-cHL patients were also compared to matched HIV-infected controls without cHL. IL10, IL6, and BAFF levels were higher in HIV-cHL patients than in controls (*p* < 0.0001, *p* = 0.002, and *p* < 0.0001, respectively). Cytokine levels increased in patients with advanced-stage lymphoma compared to those with limited-stage (*p* = 0.002, *p* = 0.03, and *p* = 0.01, respectively). Cytokine levels significantly decreased following HIV-cHL diagnosis and treatment. Blood counts of whole B-cells were similar in HIV-cHL patients and controls, but the distribution of B-cell subsets was different with higher ratios of naive B-cells over memory B-cells in HIV-cHL patients. Blood accumulation of naive B-cells was more marked in patients with advanced cHL stages (*p* = 0.06). During the follow-up, total B-cell counts increased (*p* < 0.0001), and the proportion of naive B-cells increased further (*p* = 0.04). Together the results suggest that in HIV-infected patients, cHL is associated with a particular B-cell-related environment that includes increased production of B-cell-activating cytokines and altered peripheral distribution of B-cell subsets. This B-cell-related environment may fuel the process of tumorigenesis.

## 1. Introduction

Classical Hodgkin Lymphoma (cHL) has particular epidemiological, clinical, pathological, and virological features. It accounts for 10% of all lymphomas, and it is the most common B-cell-derived tumor in patients under 20 years old [1]. cHL incidence in patients with HIV infection largely exceeds that observed in the general population. The standardized incidence ratios (SIRs) are 5- to 30-fold higher than in the general population [2,3,4]. SIR for cHL are significantly elevated in all strata of sex and HIV-transmission categories [2,3,4]. However, epidemiological data suggest that cHL risk among HIV-infected patients is higher at moderate than at severe immunodeficiency levels [5].

cHL risk was also shown to increase following immune recovery due to combined antiretroviral therapy (cART) [2]. HIV infection is associated with chronic B-cell activation [6], even in patients on prolonged cART, which may contribute to cHL occurrence [7]. Indeed, immune parameters such as increased levels of B-cell activating cytokines including interleukin (IL)10, IL6 and B-cell activating factor (BAFF), or serum markers associated with B-cell activation (CRP, sCD30, sCD27, sCD23, and free immunoglobulin light chains), positively correlate with the incidence of cHL [8,9,10,11]. The peripheral B-cell compartment is also affected during cHL and a lower blood B-cell count has been associated with a poor prognosis [12,13,14]. Peripheral B-cells are tightly regulated by the cytokine milieu. Therefore, tumor-related factors could modify the peripheral B-cell compartments [15].

Here, we examined immunological markers associated with B-cell activation, including B-cell activating cytokines, immunoglobulin levels and the peripheral distribution of B-cell subsets in HIV-cHL patients.

## 2. Patients and Methods

### 2.1. Patient Populations

This study is an ancillary study of the prospective French Cohort of HIV-related lymphomas (French National Agency for Research on AIDS and Viral Hepatitis ANRS-CO16 LYMPHOVIR cohort) [16,17,18]. This cohort enrolled consecutive HIV-infected patients with newly diagnosed non-Hodgkin lymphoma (*n* = 123) and cHL (*n* = 83) in 22 French centers between 2008 and 2015. The histological, clinical, and outcome characteristics of HIV-cHL patients have been described in previous reports [16,18]. The most frequent histology was mixed cellularity (71%) followed by nodular sclerosis (14%). Tumor EBV status was investigated by expression of EBV-latent membrane protein-1 by immunohistochemistry and/or EBV-encoded RNA 1 by in situ hybridization. It was positive in 42% of cases. As stated in the initial report [16], patients with clinical stage I or II received first-line ABVD (3 to 4 cycles) followed by radiotherapy. All but three patients with stage III or IV cHL also received first-line therapy with standard or modified ABVD regimens. Four patients underwent autologous bone marrow transplantation after relapse.

Blood samples were collected at cHL diagnosis then every six months for 48 months. In the present study, we compared blood samples collected at cHL diagnosis to those collected up to 24 months after diagnosis. Moreover, serum samples drawn for routine HIV infection follow-up between one and three years prior to HIV-cHL diagnosis were obtained from clinical virology laboratories. A subpopulation of the Lymphovir cohort was selected for a case/control analysis. Patients were matched according to age, sex, viral load, and CD4 T-cell counts to HIV-1 positive controls recruited in two hospitals (Kremlin Bicêtre and Antoine Béclère Hospitals). This group was assigned for further case-control analysis of B-cell subsets and serum markers related to B-cell activation.

### 2.2. Immunological Analyses

Serum studies: Blood samples were collected from the enrolled patients and controls in dry tubes. Sera were aliquoted and stored at −80 °C until use. IL10, IL6, and BAFF serum levels were determined using commercially available ELISA kits (Quantikine^®^ Colorimetric Sandwich ELISA Kits, all from R&D Systems, Minneapolis, MS, USA). Serum IgG, IgA, and IgM concentrations (g/L) were determined by nephelometry (BN ProSpec^®^, Siemens Healthcare Diagnostics, Erlangen, Germany).

Flow cytometry analysis: All flow cytometry analysis were performed at the laboratory of Immunology of Bicêtre Hospital (HHC, RK, YT). Blood samples collected on EDTA tubes were analyzed within 24 h after shipment. All antibodies used are listed in Supplementary Methods (Supplementary Tables S1 and S2). Absolute counts of CD3 T-cells, CD4 and CD8 T-cells, B and NK-cells were determined by using the BD Multitest™ 6-Color TBNK Reagent, with BD Trucount™ tubes (BD Biosciences, San Jose, CA, USA). Flow cytometry analysis was performed by using a FACSCanto cytometer (BD Biosciences).

### 2.3. Statistical Analyses

We used the McNemar test to compare proportions, and the Wilcoxon paired test to compare quantitative variables between HIV-cHL patients and matched controls, and the quantitative measures at diagnosis and at two years. All *p*-values are reported for two-tailed tests with a significance threshold of 0.05. All analyses were performed with SAS 9.4^®^ software.

## 3. Results

### 3.1. Characteristics of Patients at cHL Diagnosis

HIV-cHL patient populations (Table 1): Among the 83 HIV-cHL patients enrolled in the ANRS-CO16 LYMPHOVIR cohort between 2008 and 2015, 38 patients had a complete 24-month immunological follow-up and were analyzed in the longitudinal analysis. Among them, at cHL diagnosis, 33 patients had an HIV-1 RNA plasma load below 200 copies/mL. Median CD4^+^ T-cell count was 267 cells/μL (interquartile range (IQR) (125–510)). Eight patients had a localized lymphoma and 30 patients had a widespread (stage III and IV) disease. Among those 38 patients, seven had pre-diagnosis sera that could be analyzed. Those seven patients had a serum HIV-1 RNA load below 200 copies/mL and advanced stage of cHL.

Case control study: Fourteen HIV-cHL patients had available HIV-infected matched controls. Characteristics of HIV-cHL patients and controls are shown in Table 2. No difference between HIV-cHL patients and controls was observed for sex (*p* = 1), HIV plasma viral loads (*p* = 1), serum IgG levels (*p* = 0.31) and median CD4+ T-cell counts (*p* = 0.39). Although cases and controls were age-matched, HIV-cHL patients were younger than controls (median of 46 years, IQR (41–52) vs. 51.5 years (44–55), *p* = 0.0006). The age ranged between 33 to 56 years and 39 to 58 years in HIV-cHL patients and controls, respectively. This difference was due to delays in including controls after their selection and to the rarity of controls with the same characteristics as cases.

### 3.2. Increased Serum Levels of Cytokines Related to B-Cell Activation (IL10, IL6 and BAFF) in HIV-cHL Patients at cHL Diagnosis

**Figure 1:**
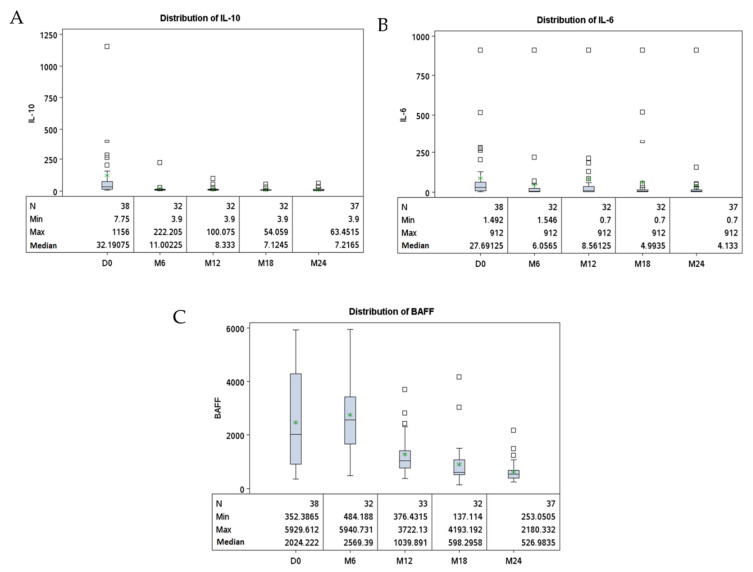
Longitudinal distribution of serum markers in HIV-positive cHL patients. Legend: Each panel represents the distribution of serum levels (pg/mL) of IL10 (**A**), IL6 (**B**) and BAFF (**C**) collected every 6 months during 24 months.*: mean; D: day; M: month.

We analyzed serum levels of IL10, IL6 and BAFF, three cytokines associated with B-cell activation. When compared to controls, at diagnosis, HIV-cHL patients had consistently higher serum levels of IL10 (*p* < 0.0001), IL6 (*p* = 0.002) and BAFF (*p* < 0.0001). Those higher cytokine levels had no impact on immunoglobulin production. Indeed, serum levels of IgG, IgA or IgM, did not differ significantly between HIV-cHL patients and controls (Table 2). At diagnosis, IL10, IL6 and BAFF cytokine serum levels were higher in cHL patients with advanced-stage (Ann Arbor III-IV) lymphoma compared to patients with localized disease (*p* = 0.002, *p* = 0.03 and *p* = 0.01, respectively). In contrast, there was no significant difference in IgG, IgA or IgM serum levels according to clinical stage (*p* = 0.11, *p* = 0.94 and *p* = 0.76) (Table 3). In the 38 patients followed longitudinally, IL10, IL6 and BAFF serum levels decreased during the 24 months following cHL diagnosis (*p* < 0.0001, *p* = 0.02 and *p* < 0.0001), respectively (Table 4 and Figure 1).

In the 7 patients with available pre-cHL diagnostic serum samples (Supplementary Tables S1 and S2), the median IL10 serum level was lower prior to cHL diagnosis (*p* = 0.02). IL6 and BAFF serum levels also tended to be lower before cHL diagnosis, although the differences were not significant (*p* = 0.22 and *p* = 0.30, respectively).

### 3.3. Altered Peripheral B-Cell Compartment in HIV-1 Infected-Patients Developing cHL

At cHL diagnosis, HIV-cHL patients and their controls showed similar blood counts of CD4^+^ T-cells (*p* = 0.39), CD8^+^ T-cells (*p* = 0.33), CD3^−^CD56^+^CD16^+^ NK-cells (143.5 vs. 247.5 cells/μL, *p* = 0.17) and total CD19^+^ B-cells (*p* = 0.91) (Table 2). However, HIV-cHL patients had significantly increased proportions of CD27^−^IgD^+^ naive B-cells compared to controls (*p* = 0.0005). Conversely, the proportion of CD27^+^IgD^−^ memory B-cells was lower in HIV-cHL cases than in controls (*p* = 0.005), while the frequencies of B-cells with a marginal zone-like phenotype (CD27^+^IgD^+^) did not differ significantly between HIV-cHL patients and controls (*p* = 0.62) (Table 2). It is worth noting that the accumulation of CD27^−^IgD^+^ naive B-cells in the blood tended to be more marked in patients with advanced cHL stages (*p* = 0.06) (Table 3).

**Figure 2:**
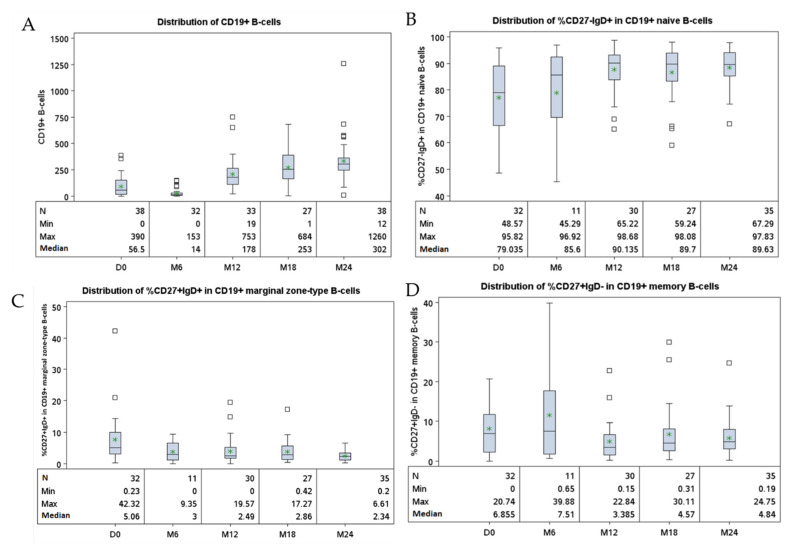
Longitudinal distribution of lymphocyte subsets in HIV- cHL patients. Legend: The panels represent the distribution of CD19^+^ cell counts (cells/μL) (**A**) and, among total B-cells, the proportions of naive (**B**), marginal zone type (**C**) and memory cells (**D**) collected every 6 months for 24 months starting from cHL diagnosis. *: mean; D: day; M: month.

At 24 months after HIV-cHL diagnosis, there was an increase in absolute counts of B-cells (<0.0001), CD4^+^ T-cells (*p* = 0.005), CD8^+^ T-cells (*p* = 0.04) and NK-cells (*p* = 0.0002) (Supplementary Figure S1). Moreover, the proportion of CD27^−^IgD^+^ naive B-cells among total B-cells increased as compared to values observed at diagnosis (*p* = 0.04), while the proportion of CD27^+^IgD^+^ marginal zone-type B-cells decreased (*p* = 0.02). The proportion of CD27^+^IgD^−^ memory B-cells among total B-cells did not vary significantly 24-months after diagnosis (*p* = 0.32) (Table 4 and Figure 2). When comparing the cases at 24 months after diagnosis and controls, the proportion of naive B-cells was still higher in cases than in controls. On the contrary, cases had lower proportion of memory B-cells at 24 months after diagnosis than controls (Supplementary Table S2).

## 4. Discussion

HIV infection is associated with important chronic B-cell activation and initial follicular hyperplasia. Persistent chronic activation of B-cell compartment can contribute to increased cHL occurrence in HIV-infected patients. Here, we analyzed in a cohort of HIV-cHL patients the serum profile of cytokines associated with B-cell activation and the composition of the blood B-cell pool. We provided evidence that HIV-cHL is associated with increased levels of IL10, IL6 and BAFF. These cytokines correlated with tumor burden and decreased during follow-up. While HIV-cHL patients and their matched controls had similar total CD19^+^ B-cell blood counts at cHL diagnosis, HIV-cHL patients had lower proportions of circulating CD27^+^IgD^−^ memory B-cells at cHL diagnosis. This was associated with a higher proportion of CD27^−^IgD^+^ naive B-cells. Moreover, the peripheral expansion of naive B-cells in HIV-cHL patients was more marked in the advanced stages of the disease, correlating with cHL tumor burden.

cHL tumors are associated with a complex local cytokine milieu at the tumor site, and to elevated cytokine levels in serum. IL10 that strongly inhibits cell-mediated immunity and inflammation, and promotes plasma cell differentiation, is known to be produced by cHL tumor cells [19]. Elevated serum levels of IL10 before diagnosis of cHL, that has been already reported [6,8] was confirmed in our study. HIV is also known to induce IL10 production by monocytes [20,21] and NK-cells [22]. In our study, IL10 appears to correlate with tumor burden and to decrease during the follow up. IL6 is a potent pleiotropic cytokine with both pro-inflammatory as well as anti-inflammatory/regulatory functions [23,24,25]. IL6 induces proliferation and maturation of B-cells towards antibody-producing cells but it also regulates hematopoiesis, supporting early hematopoietic progenitor growth [26,27,28]. Notably, IL6 signaling appears to be involved in B-cell lymphomagenesis including cHL [29], and serum IL6 levels were found to be higher in the months preceding cHL [8]. Furthermore, HIV-1 infection is associated with increased IL6 production [6]. In our study, IL6 levels were higher in HIV-cHL cases than in HIV controls. Patients with advanced disease had higher levels of IL6, suggesting increased production by the tumor and/or by the immune effectors activated by the tumor. Autocrine or paracrine IL-6 signaling may fuel the tumor growth. IL6 levels decreased after cHL treatment to pre-diagnosis levels, pointing out the relation between the size of the tumor and the levels of IL-6. BAFF is a member of the TNF family of cytokines, which promotes the survival and differentiation of B-cells [30,31]. Increase in BAFF levels occurs during HIV infection [32,33,34], as well as in B-cell lymphoproliferative disorders [35,36], such as B chronic lymphocytic leukemia [37], non-Hodgkin lymphoma [38], multiple myeloma [39] and Waldenström macroglobulinemia [40]. cHL tumors were also shown to produce BAFF both by Reed-Sternberg cells and by infiltrating myeloid cells, leading to an enhanced tumor cell survival via NF-κB activation [41]. Similarly to IL6 and IL10 [42], circulating BAFF levels correlate with complete remission rate, overall survival and progression-free survival in AIDS-associated NHL [43]. In our study, serum levels of BAFF diminished during the follow-up along with the restoration of B-cell count. This might be due to a correlation with the tumor burden, and to regulation mechanisms involving B-cell-related factors such as the number of B-cells, and the density of expression of BAFF-binding receptors [44]. Overall, these cytokines that act at the tumor microenvironment level may also have a systemic impact, especially when acting in concert, and may influence B-cell subset composition, activation and trafficking.

Circulating B-cells represent an important part of the whole body B-cell pool [45,46]. CD27^−^IgD^+^ naive B-cells represent a major part of blood B-cells [47]. CD27^+^IgD^−^ memory B-cells are immunoglobulin class-switched B lymphocytes expressing surface IgG or IgA [48,49]. In contrast, CD27^+^IgD^+^ marginal-zone type B-cells mainly express surface IgM are critically involved in the response to T-independent Ags [50]. The finding of CD27^+^IgD^−^ memory B-cells lymphopenia at cHL diagnosis raises the hypothesis of a preexisting abnormal B-cell subset distribution within peripheral B-cells in HIV-1-infected patients prone to develop cHL. Or, cHL may trigger altered redistribution or sequestration in lymphoid tissues, or selective apoptosis of the CD27^+^IgD^−^ memory B-cell subset [51]. Notably, while serum levels of all cytokines tested decreased over time, the abnormal distribution of B-cell subsets in HIV-cHL was even more pronounced 24 months after diagnosis. A significant increase in the proportion of circulating naive B-cells is observed in HIV-cHL patients at the expense of marginal zone B-cells and memory B-cells. This demonstrates that the chemotherapy regimen used for HIV-cHL did not enable recovery of a normal B-cell subset distribution patterns within peripheral B-cells.

To our knowledge, this is the first report focusing on the peripheral B-cell distribution pattern in HIV-cHL, including prospective 24 months follow-up, within a homogenous population, with available matched HIV-infected controls and pre-diagnosis serum samples. Significant limitations of this study include the limited numbers of study subjects, and the lack of correlation between EBV infection status and immunological analysis of the B-cell population. Indeed, active EBV replication can be an independent factor that strongly modulates peripheral B-cell compartment by a complex network of induced soluble factors and cell-membrane co-stimulating receptors.

## 5. Conclusions

HIV-cHL patients have an abnormal profile of cytokines that may impact B-cell activation, differentiation and survival, as shown by a significantly altered composition of the peripheral B-cell pool. A better understanding of the mechanisms underlying those abnormalities may provide clues for future therapeutic strategies of HIV-infection-associated cHL.

## Figures and Tables

**Table 1:** Characteristics of the study population at cHL diagnosis.

		Patients with Pre-Diagnosis Samples (*n* = 7)	Patients with a 24-Month Immunological Follow-Up (*n* = 38)
Age (years)			
	Median	42	43
	IQR	39–53	38–47
Gender			
	M	7	33
	F	0	5
Lymphoma stage			
	I-II	0	8
	III-IV	7	30
Histology			
	Nodular Sclerosis	1 (14.3)	3 (7.9)
	Mixed Cellularity	4 (57.1)	27 (71.1)
	Lymphocyte Predominance	0 (0.0)	1 (2.6)
	Not categorized	2 (28.6)	7 (18.4)
Immunological Markers			
IL10 (pg/mL)			
	Median	20.3	32.2
	IQR	17.5–157.1	14.5–75.4
IL6 (pg/mL)			
	Median	60.5	27.7
	IQR	6.8–266.4	8.1–60.5
BAFF (pg/mL)			
	Median	2075.1	2024.2
	IQR	585.1–4381.0	913.7–4288.9
CD4^+^ T-cells (Cells/μL of blood)			
	Median	465	380
	IQR	127–596	144–483
CD8^+^ T-cells (Cells/μL of blood)			
	Median	692	544
	IQR	410–1806	394–784
CD19^+^ B-cells (Cells/μL of blood)			
	Median	161	56
	IQR	26–196	16–153
CD3^+^CD4^+^ T-cells (Cells/μL of blood)			
	Median	487	267
	IQR	234–579	125–510
CD3^+^CD8^+^ T-cells (Cells/μL of blood)			
	Median	788	496
	IQR	427–1357	325–788
CD56^+^CD16^+^ NK-cells (Cells/μL of blood)			
	Median	160	127
	IQR	105–256	76–177

cHL: classical Hodgkin lymphoma; IQR: interquartile range; NK: natural killer.

**Table 2:** Characteristics of HIV-cHL patients with matched controls at cHL diagnosis.

		Populations		*p*-Values *
		cHL Patients (*n* = 14)	Controls (*n* = 14)	
Age				0.0006
	Median	46	51	
	IQR	41–52	44–55	
Sex				1
	M	12	12	
	F	2	2	
HIV viral Load (copies/mL)				1
	≤200	13	12	
	>200	1	2	
Serum Markers				
IgG levels (g/L)				0.31
	Median	13.2	13.3	
	IQR	12.1–16.5	10.1–15.6	
	*n*	14	14	
IgA levels (g/L)				0.19
	Median	3.2	2.2	
	IQR	1.6–4.0	1.7–2.6	
	*n*	14	14	
IgM levels (g/L)				0.49
	Median	0.8	0.6	
	IQR	0.5–1.1	0.4–1.0	
	*n*	14	14	
IL10 (pg/mL)				<0.0001
	Median	22.4	4.5	
	IQR	11.2–54.4	3.9–7.5	
	*n*	14	14	
IL6 (pg/mL)				0.002
	Median	23.5	2.1	
	IQR	5.2–128.7	0.9–9	
	*n*	14	14	
BAFF (pg/mL)				<0.0001
	Median	898.9	398.8	
	IQR	720.2–1790.3	323.2–445.4	
	*n*	14	14	
Lymphocytic subpopulations				
CD45^+^ Lymphocytes				0.63
(cells/μL)	Median	1488	1629	
	IQR	966–2570	1203–2086	
	*n*	14	14	
CD3^+^CD4^+^ T-cells				0.39
(cells/μL)	Median	498.5	417.5	
	IQR	328–579	386–558	
	*n*	14	14	
CD3^+^CD8^+^ T-cells				0.33
(cells/μL)	Median	616.5	646.5	
	IQR	415–1208	431–741	
	*n*	14	14	
CD19^+^ B-cells				0.91
(cells/μL)	Median	165	170.5	
	IQR	87–210	125–222	
	*n*	14	14	
%CD27^−^IgD^+^ CD19^+^ naive B-cells				0.0005
	Median	76.2	53.1	
	IQR	64.7–89.1	38.3–62.2	
	*n*	12	12	
%CD27^+^IgD^+^ in CD19^+^ marginal zone-type B-cells				0.62
	Median	5.5	11.5	
	IQR	4.1–20.5	5.6–14.1	
	*n*	12	12	
%CD27^+^IgD^−^ in CD19^+^ memory B-cells				0.0005
	Median	6.7	25.2	
	IQR	1.8–11.5	20.5–32.2	
	*n*	12	12	
CD56^+^CD16^+^ NK-cells				0.17
(cells/μL)	Median	143.5	247.5	
	IQR	81–258	125–406	
	*n*	14	14	

* *p* values were determined by the McNemar test (categorical variables) or the Wilcoxon signed-rank test (continuous variables). cHL: classical Hodgkin lymphoma; IQR: interquartile range; NK: natural killer.

**Table 3:** Serum markers and lymphocyte subsets in HIV-cHL patients according to lymphoma clinical stages.

		Population		*p*-Value *
		Ann Arbor I-II (*n* = 18)	Ann Arbor III-IV (*n* = 26)	
Serum Markers				
IL10 (pg/mL)				0.002
	Median	11.0	31.4	
	IQR	6.7–26.8	16.6–69.1	
	*n*	18	26	
IL6 (pg/mL)				0.03
	Median	5.1	23.9	
	IQR	2.7–30.9	10.8–60.4	
	*n*	18	26	
BAFF (pg/mL)				0.01
	Median	777.1	1039.6	
	IQR	471.1–1180.9	913.7–2338.0	
	*n*	18	26	
IgG levels (g/L)				0.11
	Median	12.1	16.2	
	IQR	10.75–16.5	12.8–18.8	
	*n*	16	24	
IgA levels (g/L)				0.94
	Median	3.0	2.6	
	IQR	1.9–3.7	1.8–3.7	
	*n*	16	24	
IgM levels (g/L)				0.76
	Median	0.8	0.7	
	IQR	0.7–1.0	0.4–1.2	
	*n*	16	24	
Lymphocytic subpopulations				
CD3^+^CD4^+^ T-cells				0.50
(cells/μL)	Median	509	462	
	IQR	380–581	349–623	
	*n*	18	26	
CD3^+^CD8^+^ T-cells				0.71
(cells/μL)	Median	614	672	
	IQR	514–891	394–797	
	*n*	18	26	
CD19^+^				0.30
(cells/μL)	Median	137	83	
	IQR	49–222	42–167	
	*n*	16	25	
% CD27^−^IgD^+^ naive B-cells				0.06
in CD19^+^	Median	66.0	84	
	IQR	48.6–82.2	67.1–92.4	
	*n*	13	21	
% CD27^+^IgD^+^ marginal zone-type				0.10
B-cells in CD19^+^	Median	14.4	4.01	
	IQR	3.0–21.1	2.3–7.6	
	*n*	13	21	
% CD27^+^IgD^−^ memory				0.21
B-cells in CD19^+^	Median	10.6	5.9	
	IQR	6.8–19.9	2.1–5.9	
	*n*	13	21	
CD56^+^CD16^+^ NK-cells				
(cells/μL)	Median	151	129	0.45
	IQR	82–270	80–167	
	*n*	16	25	

* *p* values were determined by the Wilcoxon signed-rank test. cHL: classical Hodgkin lymphoma; IQR: interquartile range; NK: natural killer.

**Table 4:** Comparison of lymphocyte subsets and serum marker levels collected at HIV- cHL diagnosis and 24 months after.

		At cHL Diagnosis (*n* = 38)	At 24 Months (*n* = 38)	*p*-Value *
Serum Markers				
IL10 (pg/mL)				<0.0001
	Median	36.4	7.2	
	IQR	17.5–75.4	4.2–13.0	
	*n*	37	37	
IL6 (pg/mL)				0.0002
	Median	28.0	4.1	
	IQR	8.1–60.5	1.9–14.4	
	*n*	37	37	
BAFF (pg/mL)				<0.0001
	Median	2043.8	527.0	
	IQR	932.2–4288.9	390.8–674.6	
	*n*	37	37	
IgG levels (g/L)				<0.0001
	Median	14.1	11.8	
	IQR	11.3–18.0	10.0–12.9	
	*n*	37	37	
IgA levels (g/L)				
	Median	2.9	2.5	0.0003
	IQR	1.6–4.3	1.7–3.0	
	*n*	37	37	
IgM levels (g/L)				0.03
	Median	0.6	0.5	
	IQR	0.4–1.1	0.4–0.8	
	*n*	37	37	
Lymphocytic subpopulations				
CD3^+^CD4^+^ T-cells				<0.0001
(cells/μL)	Median	267	490	
	IQR	125–510	330–681	
	*n*	38	38	
CD3^+^CD8^+^ T-cells				0.01
(cells/μL)	Median	496	787	
	IQR	325–788	575–1027	
	*n*	38	38	
CD19^+^ B-cells				<0.0001
(cells/μL)	Median	56	302	
	IQR	16–153	247–362	
	*n*	38	38	
%CD27^−^IgD^+^ in CD19^+^ naive B-cells				<0.0001
	Median	78.4	89.6	
	IQR	66.0–86.7	84.9–93.7	
	*n*	31	31	
%CD27^+^IgD^+^ in CD19^+^ marginal zone-type B-cells				<0.0001
	Median	5.1	2.3	
	IQR	3.0–10.7	1.1–3.4	
	*n*	31	31	
%CD27^+^IgD^−^ in CD19^+^ memory B-cells				0.01
	Median	6.9	4.8	
	IQR	2.4–11.7	3.2–8.1	
	*n*	31	31	
CD56^+^CD16^+^ NK-cells				<0.0001
(cells/μL)	Median	127	202	
	IQR	76–177	106–302	
	*n*	38	38	

* *p* values were determined by Wilcoxon signed-rank test for paired samples. cHL: classical Hodgkin lymphoma; IQR: interquartile range; NK: natural killer.

## Data Availability

The dataset is owned by ANRS (France REcherche Nord&Sud Sida-hiv Hépatites), an autonomous agency within Inserm. Data requests may be submitted to the data monitoring and analysis centre of the study and must be approved by the French computer watchdog authority, la Commission Nationale de l’Informatique et des Libertés (CNIL). Data requests may be sent to Lambert Assoumou (lambert.assoumou@iplesp.upmc.fr).

## Supplementary Materials

The following are available online at https://www.mdpi.com/article/10.3390/cancers14010128/s1. Table S1: Comparison of serum marker levels collected at HIV-cHL diagnosis and one to three years before diagnosis of cHL. Table S2: Comparisons of the immunological characteristics of patients 24 months after HIV-cHL diagnosis with their controls at baseline. Figure S1: Longitudinal evolution of TCD4, TCD8 and NK lymphocytic subpopulations in HIV-positive cHL patients.
