# Supplementary material for: Increased Production of B-Cell Activating Cytokines and Altered Peripheral B-Cell Subset Distribution during HIV-Related Classical Hodgkin Lymphoma
Source: Cancers (Basel). 2021 Dec 28;14(1):128. doi: 10.3390/cancers14010128 (PMC8750095; doi:10.3390/cancers14010128)
Supplement: Supplementary file 1 [file cancers-14-00128-s001.zip › cancers-1475163-supplementary.pdf]

cancers MDPI

Article

Increased Production of B-Cell Activating Cytokines and Altered Peripheral B-Cell Subset Distribution during HIV-Related Classical Hodgkin Lymphoma

Raphael Lievin * and Houria Hendel-Chavez *, Aliou Balde, Remi Lancar, Michele Algarte Genin, Roman Krzysiek, Dominique Costagliola, Lambert Assoumou, Yassine Taoufik *, Caroline Besson *

Supplementary Methods:

Immunological analyses: The following antibodies were used to assess B-cell subsets: anti CD45 (PerCP), anti-CD19 (PerCP), anti-IgD (FITC), anti-CD27 (APC), anti-BAFFR (PE), anti-CD38 (PE), anti-IgM (FITC), anti-CD21 (FITC, all from BD Biosciences). In addition, anti-CD3 (FITC), anti-CD4 (APC), anti-CD8 (FITC), anti-CD16 and anti-CD56 (PE) MoAb were used to analyze T and NK-cells populations (all from BD Biosciences).

To analyze the absolute values of T and NK populations, the following antibodies were used with 2 premixes (BD Biosciences):

(1) Anti-CD45 (PerCP), anti-CD3 (FITC), anti-CD4 (APC) and anti-CD8 (PE)
(2) anti-CD45 (PerCP), anti-CD3 (FITC), anti-CD19 (APC) and anti-CD16-56 (PE)

To calculate the % of B-cell subsets, 3 premixes were used:

(1) anti-CD19 (PerCP), anti-IgD (FITC), anti-BAFF-R (PE), anti-CD27 (APC)
(2) anti-CD19 (PerCP), anti-IgM (FITC), anti-CD38 (PE)
(3) anti-CD19 (PerCP), anti-CD21 (FITC), anti-CD38 (PE)

Table S1. Comparison of serum marker levels collected at HIV- cHL diagnosis and one to three years before diagnosis of cHL.

		Before diagnosis (N = 7)	At baseline (N = 7)	P-values*
IL-10 (pg/mL)				**0.02**
	Median	9.8	25.5	
	IQR	5.9 – 21.8	17.5 – 80.7	
IL-6 (pg/mL)				0.22
	Médiane	5.4	33.5	
	IQR	3.0 – 13.2	4.7 – 63.3	
BAFF (pg/mL)				0.30
	Médiane	553.0	960.6	
	IQR	447.5 – 942.5	514.0 – 2075.0	

* P values were determined by Wilcoxon signed-rank test for paired samples.

Table S2. Comparisons of the immunological characteristics of patients 24 months after HIV-cHL diagnosis matched with their controls at baseline.

		Populations		P-values*
		cHL patients at 24 months (N=11)	Controls at baseline (N=11)	
Serum Markers				
IgG levels (g/L)				**0.04**
	Median	10.5	14.1	
	IQR	9.6-12.4	10.3-16.5	
	N	11	11	
IgA levels (g/L)				0.95
	Median	2.5	2.2	
	IQR	1.3-3.2	1.7-2.6	
	N	11	11	
IgM levels (g/L)				0.38
	Median	0.6	0.8	
	IQR	0.5-0.7	0.4-1.3	
	N	11	11	
IL-10 (pg/mL)				**0.02**
	Median	7.2	4.5	
	IQR	4.6 – 13.0	3.9 – 7.5	
	N	11	11	
IL-6 (pg/mL)				0.63
	Median	3.5	2.3	
	IQR	2.0 – 4.9	0.8 – 9.0	
	N	11	11	
BAFF (pg/mL)				0.07
	Median	605.5	426.0	
	IQR	354.7 – 730.8	335.8 – 531.3	
	N	11	11	
		Lymphocytic subpopulations		
CD45+ Lymphocytes (absolute values, cells/µL)				0.15
	Median	2526	1752	
	IQR	1085 – 2975	1546 – 2178	
	N	11	11	
CD3+CD4+ T-cells (absolute values, cells/µL)				0.10
	Median	654	414	
	IQR	330 – 735	309 – 558	
	N	11	11	
CD3+ CD8+ T-cells (absolute values, cells/µL)				0.32
	Median	819	674	
	IQR	496 – 1216	598 – 814	
	N	11	11	
CD19+ B-cells (absolute values, cells/µL)				**0.01**
	Median	358	166	
	IQR	280 – 564	125 – 256	
	N	11	11	
%CD27-IgD+ CD19+ naive B-cells				**0.002**
	Median	90.0	51.7	
	IQR	86.6-94.0	33.7-66.6	
	N	10	10	
%CD27+IgD+ in CD19+ marginal zone-type B-cells				**0.002**

	Median	2.7	11.7	
	IQR	2.4-4.8	5.4-15.2	
	N	10	10	
%CD27+IgD- in CD19+ memory B-cells				**0.002**
	Median	4.2	26.8	
	IQR	3.0-5.2	18.2-36.0	
	N	10	10	
CD56+CD16+ NK-cells (absolute values, cells/μL)				0.97
	Median	295	302	
	IQR	57 – 565	125 – 424	
	N	11	11	

* P values were determined by the Wilcoxon signed-rank test (continuous variables). cHL: classical Hodgkin lymphoma; IQR: interquartile range; NK: natural killer

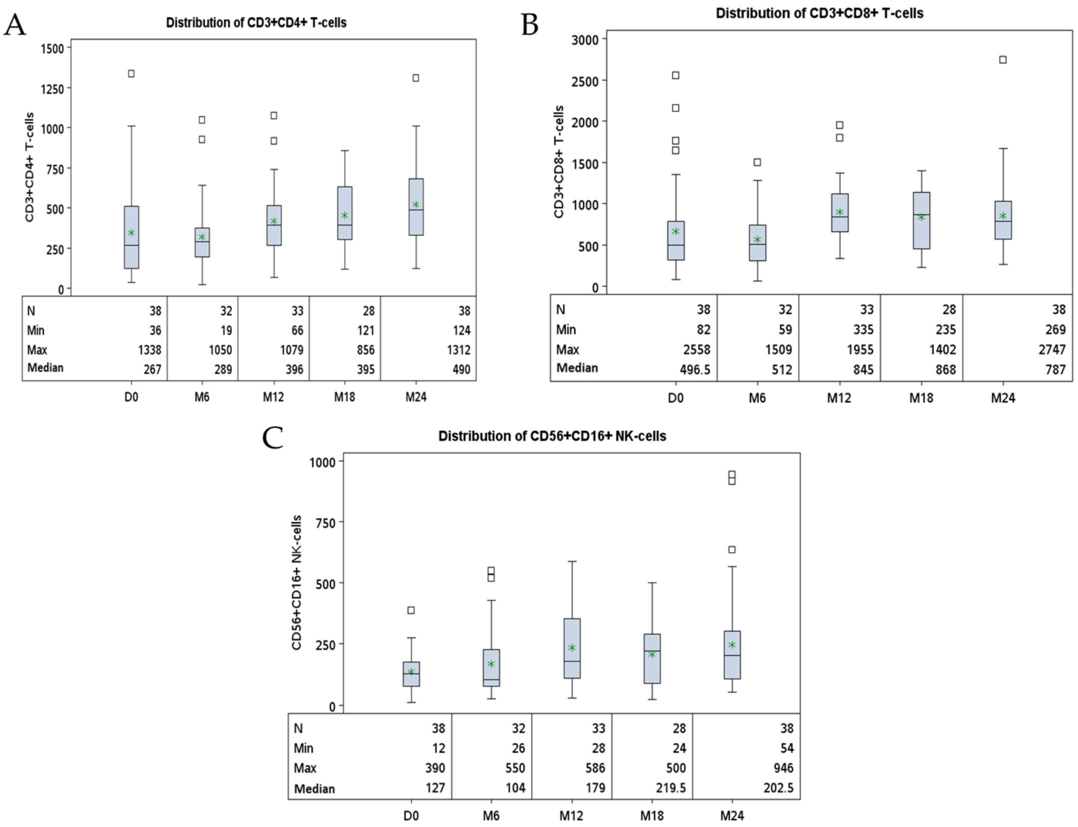

Figure S1. Longitudinal evolution of TCD4, TCD8 and NK lymphocytic subpopulations in HIV-positive cHL patients. Evolution of the counts of $CD3^+CD4^+$ T-cells (A), $CD3^+CD8^+$ T-cells (B), $CD56^+CD16^+$ NK-cells (C) every 6 months from diagnosis during 24 month.
